# Design and Evaluation of a Low-Cost Mount for Attaching a Laser Tracker’s SMR to a Robot Flange

**DOI:** 10.3390/s25010184

**Published:** 2024-12-31

**Authors:** Florian Stöckl, Silvan Müller, Marcus Strand, Markus Gardill

**Affiliations:** 1 Duale Hochschule Baden-Württemberg Karlsruhe, Robot and Human Motion (RaHM)-Lab, Erzbergerstraße 121, 76133 Karlsruhe, Germany or silvan.mueller@dhbw-karlsruhe.de (S.M.); marcus.strand@dhbw-karlsruhe.de (M.S.); 2 Fakultät 1, Brandenburgische Technische Universität Cottbus-Senftenberg, Siemens-Halske-Ring 14, 03046 Cottbus, Germany; markus.gardill@b-tu.de

**Keywords:** calibration, measurement error, repeatability, traceability

## Abstract

Robot calibration and modelling measurements are commonly performed using a laser tracker. To capture three-dimensional positions, a SMR is attached to the robot. While some researchers employ adhesive bonds for this purpose, such methods often result in inaccurate, unstable and non-repeatable SMR positioning, adversely affecting measurement precision and the traceability of research outcomes. To address these challenges, we investigated alternative methods for attaching an SMR to a robot’s flange to achieve both accuracy and repeatability. Additionally, we analysed measurement errors introduced when using a tool to attach the SMR to the flange. As a solution, we developed a 3D-printed mount designed for attachment to the flange. The mount’s accuracy was evaluated by assessing its eccentricity and the repeatability of the SMR placement. Experimental results demonstrated that the mount achieved an eccentricity radius of 0.35 mm and repeatability inaccuracies of X=0.075mm, Y=0.328mm, and Z=0.485mm. These values indicate that the mount provides sufficient accuracy to support calibration processes, ensures research traceability, and serves as a viable replacement for adhesive bonds.

## 1. Introduction

High-precision data from laser trackers are often used for robot calibration and modelling. For instance, Lattanzi et al. [1] utilised laser trackers to perform calibration based on the TCP positions of a six-axis robot arm, while Kong et al. [2] implemented a kinematic calibration procedure, dynamically updating the DH parameters using laser tracker measurements. Asif and Webb [3] noted that some researchers and institutions permanently attach SMRs, the tools used with laser trackers, to robots for dynamic calibration and adaptation during runtime. Additionally, the company RoboDK Inc. (Montreal, QC, CA) [4] employs its software in conjunction with three SMRs mounted on a tool plate at the flange to calculate the six parameters $x,\;y,\;z,\;R_{x},\;R_{y},$ and $R_{z}$ for TCP calibration using the three-dimensional coordinates of each SMR.

Various approaches to attaching SMRs to robots are described in the literature. Some researchers use metal plates with monuments for the SMRs [5,6]. In [2,4,7], monuments were fixed with with adhesive bonds to a metal plate. Ferrarini [7] introduced a monument-based attachment system that incorporated strings. Asif and Webb [3] directly bound a monument to a robot and placed an SMR into it. Other methods include using nests for SMR positioning [8,9] or magnetic monuments attached to a robot [5,10,11]. Companies like MetrologyWorks, Inc. (Blue Springs, MO, USA) offer bolt-on monuments (https://www.metrologyworks.com/products/laser-tracking-tooling-accessories/1x5-smr-nests/monuments-and-drift-nests/bolt-on-monuments/ (accessed on 23 August 2024)), but these are not robot-specific. The mismatch in screw sizes complicates their use on robot flanges, and an adapter, while a workaround, can reduce fixation accuracy. Furthermore, their bulkiness limits accessibility for smaller laboratories. Alternative approaches include the use of active targets, such as those employed by Santolaria et al. [12]. Active targets remain aligned with the laser tracker and enable the measurements of six dimensions through the provision of rotation information in addition to three-dimensional coordinates. Some papers mention the position of the SMR for the measurement. The authors either use the robot flange [2,4,5,7,13,14,15,16], the links of the robot [7] or an end-effector attached to the robot flange [9,17,18,19,20,21,22,23,24,25].

None of the reviewed papers include a standardised mount or provide detailed instructions for reconstructing the mounts used in their experiments. This lack of standardisation poses significant challenges for understanding and replicating findings and repeating experiments. In many cases, it is unclear which mount was used, the mount itself is inaccessible or expensive, or, when adhesive bonds are employed, the exact placement of the SMR remains ambiguous. Adhesive bonds further complicate repeatability in experimental measurements. When the robot is shared among multiple users in a laboratory, removing and reattaching the SMR results in substantial positional variation, as will be demonstrated in the baseline measurements in Section 3.1.

These challenges raise the questions of how an SMR can be attached to a robot’s flange to ensure accurate and repeatable measurements and what errors might rise from using an attachment tool for the SMR.

This article proposes a novel solution in the form of a 3D-printed mount designed to attach a laser tracker’s SMR to a robot flange. When combined with an SMR monument featuring a rear-side magnet, the proposed mount enables flexible, repeatable, and precise measurements.

To assess the accuracy of the proposed solution, we conducted two experiments. First, we measured the eccentricity of the designed and printed mount. Second, we evaluated the repeatability of the SMR placement. This work introduces a low-cost, accessible mount that can be adopted by a wide range of laboratories and research institutions, enhancing calibration processes, improving the traceability of research findings, and advancing experimental repeatability.

## 2. Materials and Methods

The mount for attaching a laser tracker’s SMR to a robot flange was designed to meet several key requirements. First, the mount had to position the SMR as close to the robot flange as possible to minimise the offset of the measurement from the flange, which serves as the standard TCP. This proximity ensures that the mount behaves similarly to a tool, particularly in terms of vibration characteristics, thereby producing calibration results that closely reflect real-world applications. Second, the mount was designed for the ease of fabrication using standard 3D printers, such as the Prusa i3 MK3S+ (Prusa Research a.s., Prague, Czech Republic). It was optimised to use minimal material, making it cost-effective, lightweight, and sustainable. Third, the mount complies with the ISO 9409-1-50-4-M6 standard [26] for robot flanges, excluding the directional pin, ensuring compatibility with collaborative robots such as the Universal Robots UR3e, UR5e and UR10e (Universal Robots A/S, Odense SØ, Denmark). Finally, the mount was designed to centre an SMR monument, enabling precise and repeatable fixation with a reduced eccentricity error. This feature enhances the accuracy and reliability of the measurements.

### 2.1. Design of Mount

**Figure 1:**
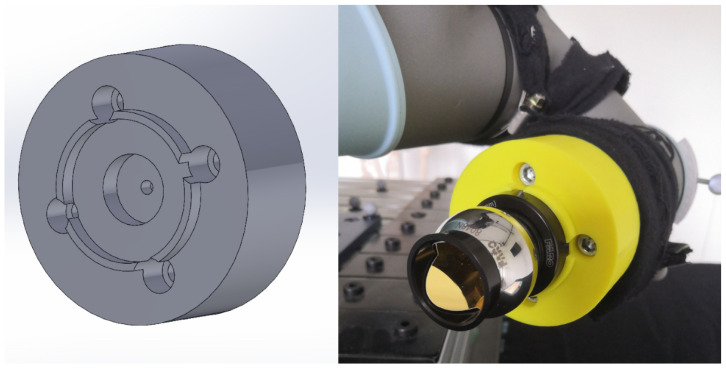
3D visualisation and application of designed mount.

Based on the specified requirements, the mount depicted in Figure 1 was designed and constructed. The 3D model and detailed construction files are publicly available on Thingiverse (3D-printable model on Thingiverse: https://www.thingiverse.com/thing:6792244 (accessed on 20 December 2024)).

**Figure 2:**
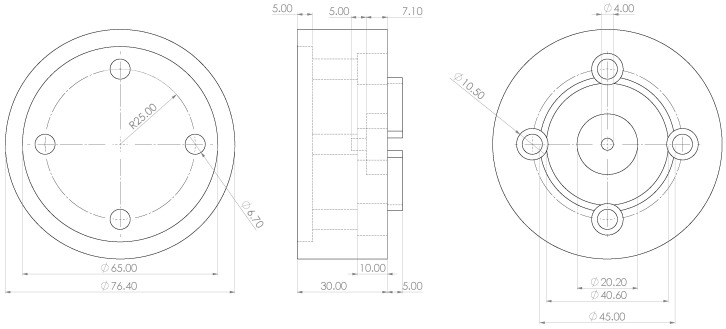
Technical drawing of designed mount with dimensions in mm.

The base of the mount is a cylinder with a diameter of 76.4 mm and a height of 30 mm. To attach the mount to a robot flange, such as that of the Universal Robots UR5e, a cylindrical recess with a diameter of 65 mm and a depth of 5 mm is incorporated into the bottom of the base cylinder. This design leaves a surrounding wall with a thickness of 5.7 mm and a height of 5 mm, ensuring a tight fit on the robot flange and preventing movement along the *x* and *y* axes. Additional dimensional details are provided in the technical drawing shown in Figure 2.

On the opposite side of the base cylinder, two cylindrical holes are included to accommodate key components. The first hole, with a diameter of 20.2 mm and a depth of 7.1 mm, holds a neodymium magnet measuring 20 × 7 mm. Behind this, a second cylindrical hole with a diameter of 4 mm and a depth of 5 mm is incorporated to house a threaded insert. This insert is embedded into the 3D-printed plastic and allows the neodymium magnet to be securely fixed in place using a screw.

In the next step, a circular ring was added to the base cylinder. This ring has an inner diameter of 40.6 mm, an outer diameter of 45 mm, a resulting wall thickness of 4.4 mm, and a height of 5 mm. Positioned at the centre of the mount, the ring, in combination with the neodymium magnet, secures an SMR monument with a magnetic back, preventing movement in the *x* or *y* direction.

The final construction step involved creating four holes for M6 screws to attach the mount to the robot flange. These holes are evenly spaced at $90^{{}^{\circ}}$ intervals along a circle with a 25 mm radius from the centre. Each hole begins with a diameter of 10.5 mm and tapers to 6.7 mm after 10 mm, with a partial overlap into the wall for the SMR monument.

Following these construction steps, the mount is compatible with ISO standard 9409-1-50-4-M6 robot flanges and accommodates SMR monuments or drift nests with dimensions ranging from 1.125–1.5 inch SMR or with a diameter of 28.6 mm. For use with robot arms featuring different flange specifications, the design can be modified by adjusting the dimensions of the mount and the hole positions, or through the use of an adapter such as the OnRobot: Flange-Adapter-Kit (OnRobot A/S, Odense SØ, Denmark) (Overview of flange adapters: https://www.memidos.com/product/onrobot-flange-adapter-kit-2/(accessed on 18 December 2024)).

### 2.2. Methods

When analysing the design of the constructed mount, primarily non-idealities due to tolerances of the 3D printing process need to be considered. All dimensions and mechanical features are referenced to the mount’s centre, meaning any slight displacement of the centre results in eccentricity throughout the mount. Similarly, misalignment between the M6 screw holes and the circular ring for the SMR introduces additional eccentricity.

**Figure 3:**
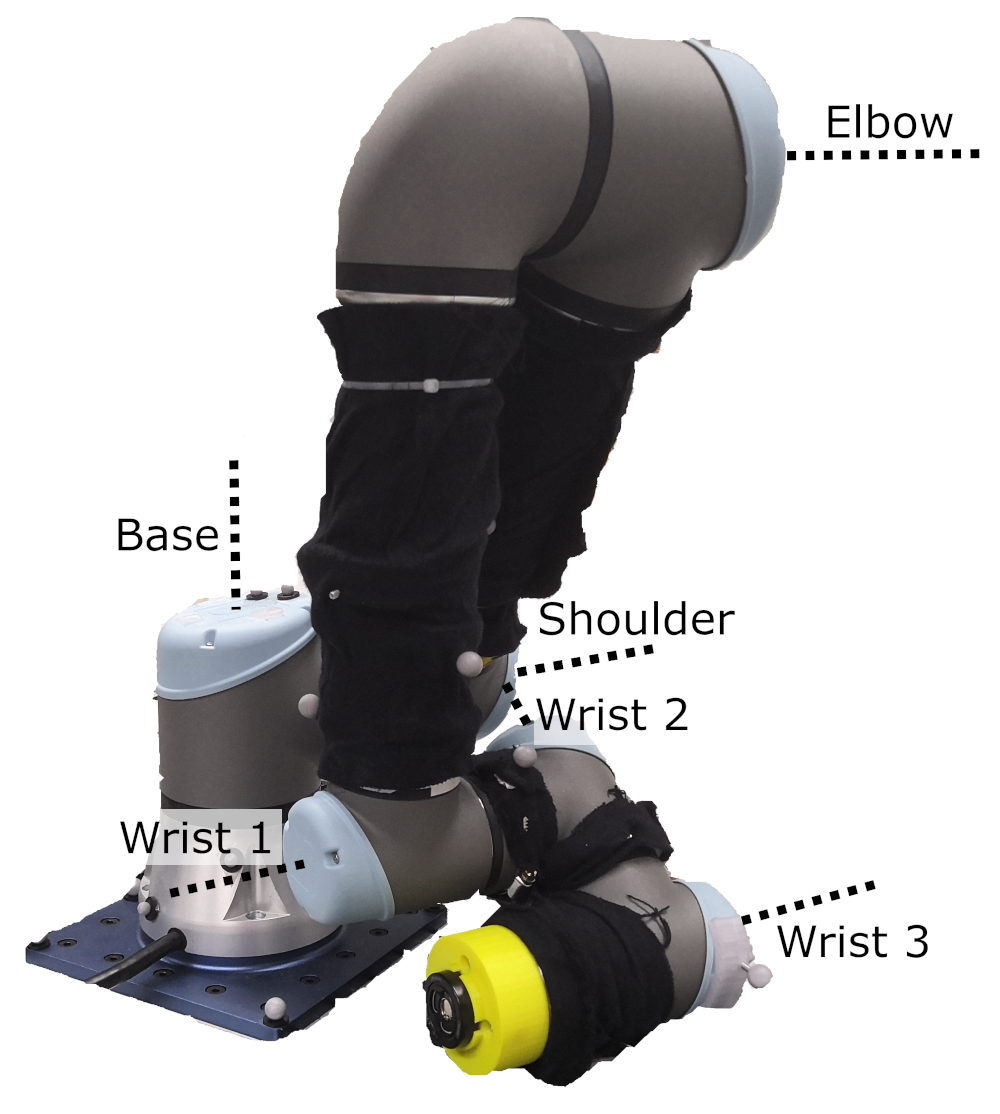
Universal Robots UR5e with labelled axes.

Two experiments were designed to evaluate inaccuracies in the mount’s design and fabrication using laser tracker measurements. The first experiment assessed potential eccentricity in the mount, while the second examined its repeatability. Both experiments were performed in the same laboratory under consistent conditions and equipment settings. For high-accuracy position measurements, a FARO Vantage E laser tracker (FARO Technologies, Inc., Lake Mary, FL, USA) was used in combination with a 1.5 inch Standard Accuracy Break Resistant SMR from FARO, featuring a gold coating, a range of 60 m, and a centring accuracy of <±7.6 μm (Technical data sheet of the FARO SMR: https://media.faro.com/-/media/Project/FARO/FARO/FARO/Resources/2021/01/15/22/46/Tech-Sheet-FARO-Laser-Tracker-Targets-ENG.pdf?rev=53837c1533274c969ac6e1d10adcd612 (accessed on 30 September 2024)). The mount was attached to a Universal Robots UR5e robot, as illustrated in Figure 3, which includes the labelling of its axes.

**Figure 4:**
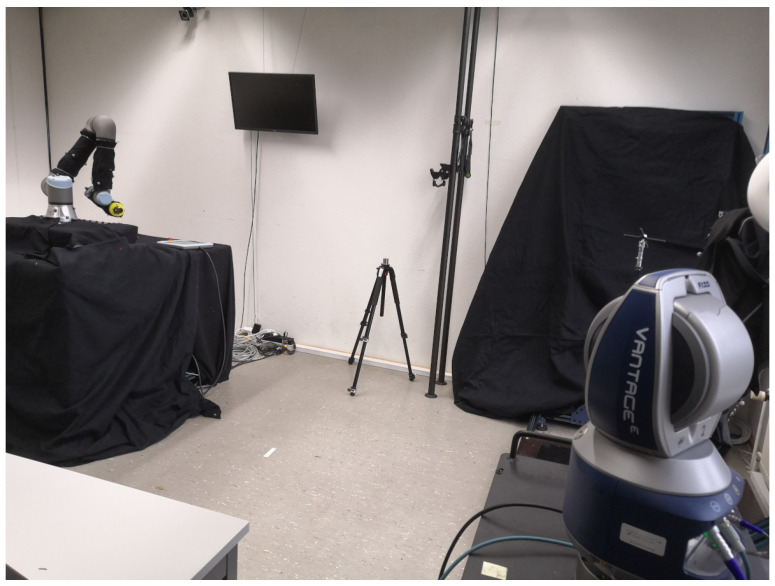
Laboratory setting for measurements of mount errors.

The measured distance between the laser tracker and the SMR was 3 m. All experiments were carried out in a laboratory on the third floor of a university building during the university’s summer holidays, so there were no students and few staff around, which reduced the movement and vibration of the floor, walls, and ceiling. During the measurements, the weather was warm and dry with a temperature of 28–30 °C and humidity of 50% to 55%. The laser tracker was always warmed up for 45 min, and a quick compensation and angle accuracy check were performed. The FARO CAM2 2024.8 software was used for data acquisition and export, collecting positional x, y, and *z* values in mm in a three-dimensional Cartesian reference frame. The origin of this frame was located at a reference point of the laser tracker, and its orientation was defined by an internal home position. In both experiments, the designed mount was attached to the robot flange facing the laser tracker, with the SMR placed inside the monument of the mount. The experimental setup is illustrated in Figure 4.

#### 2.2.1. Measurements of Eccentricity

**Figure 5:**
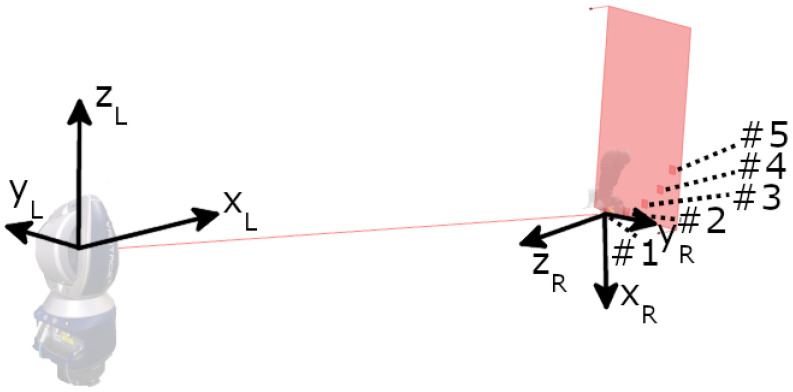
A visual representation of the experimental setup for the eccentricity measurement including the laser tracker position, the robot, their orientation vectors, and five measurement positions around the elbow joint.

To measure the mount’s eccentricity, the SMR was rotated $360^{{}^{\circ}}$ using the robot’s last joint, wrist 3, in $10^{{}^{\circ}}$ increments. At each increment, the robot stopped to allow a static laser tracker measurement. This resulted in 36 data points. In an ideal system, these points would perfectly coincide. However, any eccentricity would manifest as the points lying on a circle, with the diameter representing the eccentricity. To minimise the influence of the robot’s wrist 3, five measurement positions were obtained by rotating the elbow joint $15^{{}^{\circ}}$ four times. This experimental setup, including the laser tracker’s position and orientation, the robot’s configuration, and the measurement positions, is shown in Figure 5. The robot’s wrist 3 was aligned such that the TCP reference frame was aligned with the laser tracker’s coordinate system, which can be expressed by

(1) $$\Sigma_{L}=\left\{\vec{O_{L}},\vec{x_{L}},\vec{y_{L}},\vec{z_{L}}\right\};$$

(2) $$\Sigma_{R}=\left\{\vec{O_{R}},\vec{x_{R}},\vec{y_{R}},\vec{z_{R}}\right\};$$

(3) $$\vec{x_{L}}=-\vec{z_{R}}.$$

The eccentricity, and therefore the circular path of all subsequent measurements, should have been as small as possible. The smaller the eccentricity was, the smaller the error introduced by the designed mount in the calibration measurements of the robot was, as the alignment was irrelevant. Otherwise, the eccentricity radius was added to each measurement, affecting the overall measurement accuracy.

#### 2.2.2. Measurements of Repeatability

Repeatability was assessed using two methods. First, with the robot arm stationary, the SMR was removed from the mount and repositioned ten times. Each position was measured using the laser tracker. Second, the entire mount was removed from the robot flange, completely unscrewed, and then re-installed and tightened five times. The SMR was removed and replaced before each measurement. Each set of measurements, with repositioning ten times and remounting five times, was performed consecutively to minimise variations in environmental conditions.

## 3. Results

To assess the accuracy of the experimental setup and the FARO Vantage E laser tracker, and to evaluate the impact of the traditional method of attaching an SMR to a robot flange with an adhesive bond on measurements, baseline measurements were conducted prior to characterising the proposed mount.

All measurements are presented in mm and visualised in coordinate systems with μm scaling. The measurements include x, y and *z* values from a right-handed Cartesian coordinate system, with the laser tracker serving as the origin. For each value of x,y and *z*, the mean, median, minimum, maximum, absolute value of the difference between the minimum and maximum, and the standard deviation are displayed. The standard deviation is calculated by

(4) $$s_{N}=\sqrt{\frac{1}{N}\sum_{i=1}^{N}{\left(x_{i}-\bar{x}\right)}^{2}},$$

where *N* is the number of values, $x_{i}$ is the *i*-th value, and $\bar{x}$ is the mean of the values.

### 3.1. Baseline Measurements

**Figure 6:**
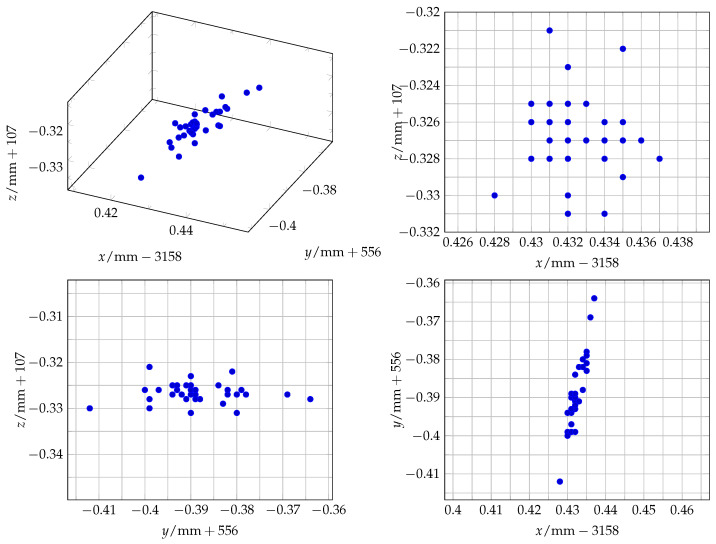
A scatter plot of the 36 static measurements which define the baseline of the laser tracker and the laboratory settings.

For the baseline measurement of the laser tracker’s accuracy, 36 measurements were taken in a series in a static position without any changes to compare the standard measurement inaccuracy in the laboratory with the values given in the laser tracker data sheet. A discussion, comparison, and classification of the measurements follows in Section 4. Figure 6 gives a visual representation of the distribution of the measurements. For a clearer and more concise presentation, the decimal part is used for the tick labels, and the integer part is added to the respective axes’ variable. In Table 1, a summarized analysis of the measured values follows.

The raw data can be found on figshare under the following link: https://doi.org/10.6084/m9.figshare.27195387.v2 (accessed on 20 December 2024).

For comparison to the designed mount, a traditional SMR fixture using an adhesive bond was analysed. In order to do this, a monument was placed as close as possible to the centre of wrist 3 of the *Universal Robots UR5e* and an SMR was placed in the monument. The eccentricity of the attachment was measured with five repetitions of a $360^{{}^{\circ}}$ rotation of wrist 3 without changing the robot’s pose with 36 measurements each. The mean eccentricity radius measured was 1.185 mm. Additionally, the repeatability of attaching the monument at the same position with an adhesive bond was tested. The monument was placed ten times as close as possible to the same position. The position of the SMR in the monument was then measured. The values are analysed in Table 2.

The raw data can be found on figshare under the following links: https://doi.org/10.6084/m9.figshare.28043018.v1 (accessed on 20 December 2024) and https://doi.org/10.6084/m9.figshare.28043048.v1 (accessed on 20 December 2024).

### 3.2. Eccentricity Measurements

**Figure 7:**
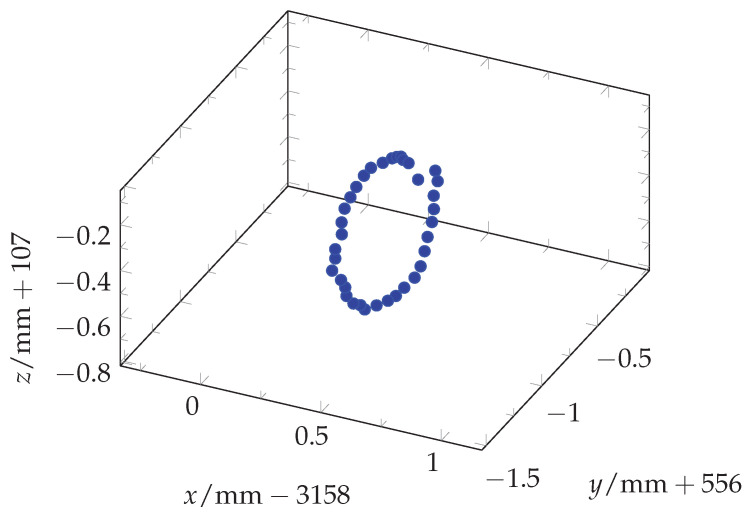
A scatter plot of the 36 measurements of one eccentricity circle (Circle 1_1).

**Figure 8:**
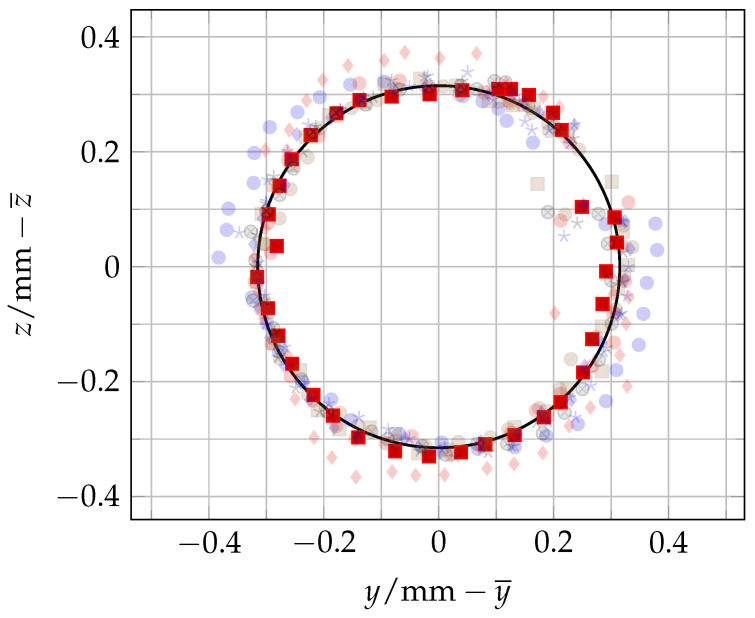
A 2D projection of the 10 eccentricity circles (represented in the plot by different symbols) with 36 measurements each, moved to the centre (0/0), and a fitted circle for Circle 1_2 with the radius $r_{\mathrm{fit}}=0.315\;\mathrm{mm}$.

Multiple measurements were taken to determine the eccentricity of the designed and 3D-printed mount. Five different poses were used and two independent $360^{{}^{\circ}}$ rotations with 36 measurements each were conducted, resulting in a total of 360 measurements. Figure 7 shows the measurements of one of those ten rotations and Figure 8 shows a projection of all 360 measurements on the yz-plane. Using the Python 3.9 package circle-fitting-3d 1.0.0 (https://github.com/CristianoPizzamiglio/circle-fitting-3d (accessed on 30 September 2024)), a circle was fitted to the 36 measurements of each of the ten rotations. The algorithm in this package is based on the blog post “Fitting a Circle to Cluster of 3D Points” by Miki [27]. It uses SVD and least squares to fit a circle to a 3D point cloud. Afterwards, the radius of each fitted circle is determined. Table 3 shows the resulting values and Figure 8 includes the fitted circle for the measurement run Circle 1_2.

The raw data of the measurements can be found on figshare under the following link: https://doi.org/10.6084/m9.figshare.27195483.v2 (accessed on 20 December 2024).

Two sources of error that affect the eccentricity measurements are the unevenness of the 3D-printed mount and the deviation from parallelism of the plane spanned by the $y_{L}$ and $z_{L}$ axes and the plane spanned by the $x_{R}$ and $y_{R}$ axes. The issue with an uneven surface is that measurements are taken on a tumbling plane when the robot’s wrist 3 is rotated, which makes it challenging and unreliable to fit a circle to the point cloud. The issue of deviation from parallelism is that the laser tracker used has an angular error that increases with distance from the laser’s origin [28]. Although the error increased only by 0.8μm/m, keeping the laser tracker’s and robot’s plane parallel was a more reliable approach. To determine the size of the errors, they were measured. Ten measurements on the monument of the mount were taken and analysed. The planarity of the printed mount was 0.014 mm and the angle between the $y_{L}$ and $z_{L}$ plane and the $x_{R}$ and $y_{R}$ plane was $0.115^{{}^{\circ}}$. In order to specify their influence on the values in Table 3, another experiment was carried out where the planes through the $y_{L}$ and $z_{L}$ axes and the $x_{R}$ and $y_{R}$ axes were orientated at an angle of $45^{{}^{\circ}}$. Five measurements of Circle 1 were made and the mean radius of these five measurements of 0.32 mm was exactly the same as the mean radius of the ten measurements in Table 3. Therefore, there was no effect of the errors on the resolution of the measurements.

The raw data can be found on figshare under the following links: https://doi.org/10.6084/m9.figshare.27468399.v1 (accessed on 20 December 2024) and https://doi.org/10.6084/m9.figshare.28025015.v1 (accessed on 20 December 2024).

In the long term, environmental influences on the mount must also be considered. As the mount is designed for calibration measurements in a laboratory setting, one relevant influence is temperature. The room temperature in a laboratory setting can vary from 17–30 °C between winter and summer months. To measure the effect of temperature on the 3D-printed mount, the mount was placed in an oven at 50 °C for 24 h. The eccentricity measurement was then repeated and the radii compared. The mean radii differed by 0.121 mm.

The raw data can be found on figshare under the following link: https://doi.org/10.6084/m9.figshare.28046144.v1 (accessed on 20 December 2024).

Another factor affecting the mount was the force exerted on the SMR during robotic arm movements. To evaluate the mount’s retention capability, the retention force was experimentally measured. The SMR was placed in the mount and subjected to an increasing pulling force until it detached. The force required for detachment was recorded. This procedure was repeated 15 times, yielding an average retention force of 18.48 N.

### 3.3. Repeatability Measurements

**Figure 9:**
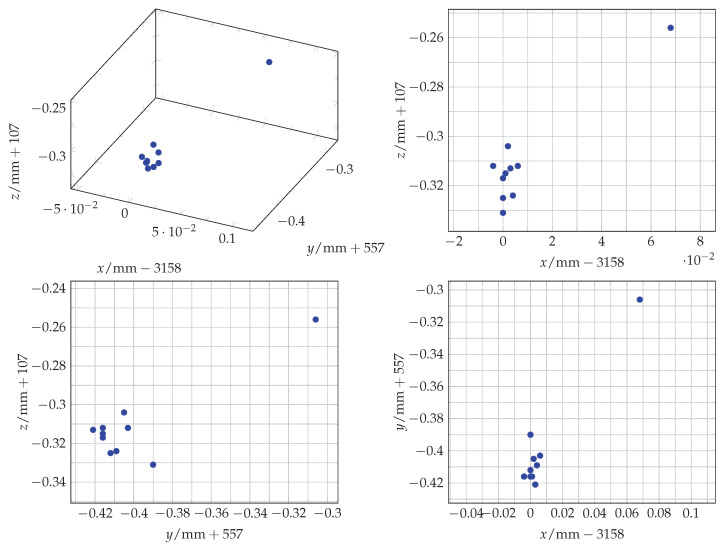
A scatter plot of the 10 measurements of placing the SMR repeatedly in the monument.

**Figure 10:**
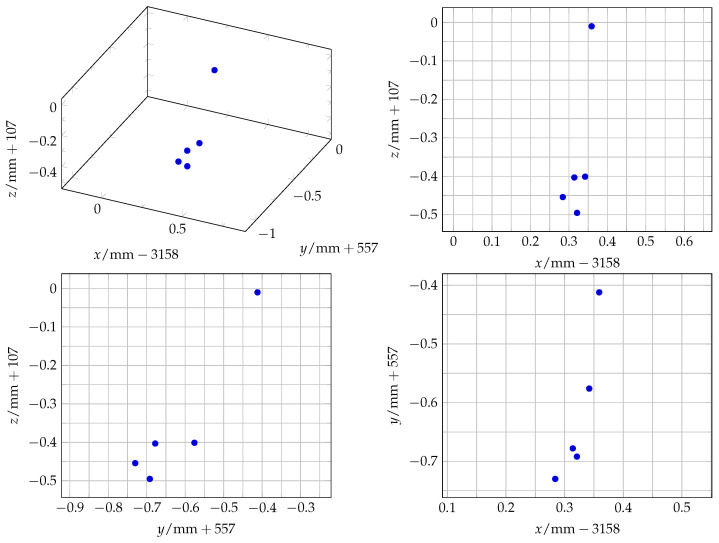
A scatter plot of the 5 measurements of mounting and dismounting the mount repeatedly.

The ten measurements of the first experiment to assess the repeatability of the SMR placement in the monument of the mount are visualised in Figure 9 and analysed in Table 4. The five values of the second experiment, where the whole mount was repeatedly mounted and dismounted, are visualised in Figure 10 and analysed in Table 5.

The raw data can be found on figshare under the following link: https://doi.org/10.6084/m9.figshare.27195540.v2 (accessed on 20 December 2024).

## 4. Discussion

This section offers a comprehensive analysis of the results presented in Section 3. It compares these results with the technical specifications, baseline measurements, and established requirements.

### 4.1. Baseline

The baseline measurements are used to determine the accuracy of the measurement setup in the laboratory with the used laser tracker FARO Vantage E, the SMR, and the environmental influence. The measured values (Table 1) are compared to the MPEs calculated with the following formulas provided by FARO in their technical data sheet for the laser tracker FARO Vantage E [28]:

- The distance error (16 μm + 0.8 μm/m) for the used laboratory settings (3 m distance between laser tracker and SMR):
  (5) $$\epsilon_{d_{\mathrm{data}\;\mathrm{sheet}}}=16\;\mu m+3\;\cdot \;0.8\;\mu m=18.4\;\mu m\equiv 0.0184\;\mathrm{mm}$$
- The angle error (20 μm + 5 μm/m) for the used laboratory settings (3 m distance between laser tracker and SMR):
  (6) $$\epsilon_{\alpha ,\beta_{\mathrm{data}\;\mathrm{sheet}}}=20\;\mu m+3\;\cdot \;5\;\mu m=35\;\mu m\equiv 0.035\;\mathrm{mm}$$

The distance and angle errors of the baseline measurement of the laser tracker and laboratory setting were calculated from the absolute value of the difference between the min and max value of the measured values for each axis:

(7) $$\epsilon_{d}=\left|\min_{i}\left(x_{i}\right)-\max_{i}\left(x_{i}\right)\right|;$$

(8) $$\epsilon_{\alpha }=\left|\min_{i}\left(y_{i}\right)-\max_{i}\left(y_{i}\right)\right|;$$

(9) $$\epsilon_{\beta }=\left|\min_{i}\left(z_{i}\right)-\max_{i}\left(z_{i}\right)\right|.$$

Table 6 shows a comparison between the theoretical measurement accuracy and the actual baseline values.

$\epsilon_{d_{\mathrm{BaseMLT}}}$ and $\epsilon_{\beta_{\mathrm{BaseMLT}}}$ are within the expected error (maximum permissible error) of the data sheet. $\epsilon_{\alpha_{\mathrm{BaseMLT}}}$ exceeds the maximum permissible error for angle errors from the data sheet ($\epsilon_{\alpha_{\mathrm{data}\;\mathrm{sheet}}}$) but only by 0.013 mm, so it is still in the same order of magnitude. And, the standard deviation $s_{N}$ of 0.0088 mm is in line with the maximum permissible error. Overall, the baseline measurements show that the laboratory environment can be used for accurate and reliable measurements.

### 4.2. Eccentricity

To contextualise the eccentricity error introduced by the designed mount, the eccentricity radii (Table 3) of the experiment in Section 2.2.1 are compared to the baseline measurements and desired accuracies mentioned in other papers [1,3,4].

The highest measured eccentricity radius was 0.353 mm. This resulted in an eccentricity diameter of 0.706 mm, which means that a measurement, which should have had exactly the same *x*, *y*, and *z* coordinates for each run, deviated in the worst case by 0.706 mm. Compared to the baseline measurement, where the largest deviation was 0.048 mm, which shows how accurate measurements could be, this was a factor of $\frac{0.706\;\mathrm{mm}}{0.048\;\mathrm{mm}}=147,08\bar{3}\approx 15$. So, instead of an accuracy in the hundredths of the millimetre range, one degree of magnitude was lost and an accuracy in the tenths of the millimetre range was achieved. But, compared to using an adhesive bond as attachment for a monument, which introduces a mean eccentricity diameter of 2.37 mm, the designed mount’s inaccuracy was lower by a factor of $\frac{0.706\;\mathrm{mm}}{2.37\;\mathrm{mm}}\approx 0.298$. Asif and Web [3] worked on an error correction in the range of 0.02 mm, which is in the range of the distance error of the FARO Vantage E laser tracker used. The radius introduced by our new tool was one order of magnitude higher. Lattanzi et al. worked on a robot positioning accuracy of up to 0.1 mm for their considered task [1]. This value was still exceeded by a factor of seven in the worst case by our mount. Finally, RoboDK developed a calibration procedure for their software which supports an accuracy of 0.05 mm for small robots and 0.15 mm for medium-sized robots [4]. Our tool is not suitable for such high-accuracy jobs. However, although the worst case of a 0.705 mm deviation is insufficient, it is not very likely. Depending on the measurement method and the trajectory, the radius is not added to the measurements, because the eccentricity does not impact the measurements. An alternative approach would be to measure eccentricity circles at each desired measurement position and use the centre of the circle as the measurement point.

One factor to consider is the structural deformation of the mount’s material, PLA, under heat. The dimensional change in the radii of 0.121 mm corresponds to $\frac{0.121\;\mathrm{mm}}{0.353\;\mathrm{mm}}=0.343\approx \frac{1}{3}$ of the total eccentricity, leading to inaccuracies in measurements. It is recommended to perform regular comparison measurements before using the mount for calibration purposes.

The designed mount is robust enough to maintain the SMR in position during robotic arm movements. With an average retention force of 18.48 N, a SMR with a mass of 144 g can theoretically withstand an acceleration of $a=\frac{F}{m}=\frac{18.48\;N}{0.144\;\mathrm{kg}}\approx 128.3\;\frac{m}{s^{2}}$. For the Universal Robots UR5e, the recommended acceleration is <$2500\;\frac{\mathrm{mm}}{s^{2}}=2.5\;\frac{m}{s^{2}}$ (Forum post by UR+ Development Support: https://forum.universal-robots.com/t/maximum-axis-speed-acceleration/13338/2 (accessed on 12 December 2024)), which is 51.32 times smaller. In addition, it can be said that the designed mount is not the limiting element but the used magnetic monument for the SMR is, which is the same as that used by other researches [5,8,9,10,11].

### 4.3. Repeatability

To provide context for the repeatability accuracies of the mount, it is compared to the baseline measurements. Therefore, the measured values for the *x*, *y* and *z* deviations are compared in Table 7. The distance and angle errors were calculated in the same way as for the baseline measurements of the laser tracker and laboratory setting using Equations (7)–(9).

As expected, the baseline measurement of the laser tracker and laboratory setting has the smallest deviations, followed by the repeatability measurement of the SMR in the monument placement and the repeatability measurement of the mounting and dismounting of the mount. The usage of an adhesive bond led to the highest deviations in repeatability. The factors of inaccuracy between the baseline (BaseMLT) and SMR in the monument placement (RepM1) are as follows:

(10) $$\frac{\epsilon_{d_{\mathrm{RepM}1}}}{\epsilon_{d_{\mathrm{BaseMLT}}}}=\frac{0.069\;\mathrm{mm}}{0.008\;\mathrm{mm}}=8.625;$$

(11) $$\frac{\epsilon_{\alpha_{\mathrm{RepM}1}}}{\epsilon_{\alpha_{\mathrm{BaseMLT}}}}=\frac{0.115\;\mathrm{mm}}{0.048\;\mathrm{mm}}=2.4958\bar{3};$$

(12) $$\frac{\epsilon_{\beta_{\mathrm{RepM}1}}}{\epsilon_{\beta_{\mathrm{BaseMLT}}}}=\frac{0.075\;\mathrm{mm}}{0.01\;\mathrm{mm}}=7.5.$$

However, despite the highest factor between the baseline and repeatability measurement, the *x*-axis still has the lowest deviation of the measurements ($\epsilon_{d}$) of all axes. A comparison between the baseline (BaseMLT) and the mounting and dismounting of the mount (RepM2) gives the following inaccuracy factors:

(13) $$\frac{\epsilon_{d_{\mathrm{RepM}2}}}{\epsilon_{d_{\mathrm{BaseMLT}}}}=\frac{0.075\;\mathrm{mm}}{0.008\;\mathrm{mm}}=9.375;$$

(14) $$\frac{\epsilon_{\alpha_{\mathrm{RepM}2}}}{\epsilon_{\alpha_{\mathrm{BaseMLT}}}}=\frac{0.328\;\mathrm{mm}}{0.048\;\mathrm{mm}}=6.8\bar{3};$$

(15) $$\frac{\epsilon_{\beta_{\mathrm{RepM}2}}}{\epsilon_{\beta_{\mathrm{BaseMLT}}}}=\frac{0.485\;\mathrm{mm}}{0.01\;\mathrm{mm}}=48.5.$$

To put the high factor for the *z*-axis into perspective, $\epsilon_{d_{\mathrm{BaseMLT}}}$ is relatively low, and the |min−max| for the *z*-value of the mounting and dismounting of the mount is relatively high compared to the *x*- and *y*-axis. However, they are still in the same order of magnitude. The difference between the *y*- and *z*-values of the SMR in monument placement and the mounting and dismounting of the mount can be explained by the microshifts of the robot during the attachment of the mount, which add up to the inaccuracy of the designed mount. To prove this, a further experiment was carried out to measure the shift of the robot during the attachment of the mount. Therefore, the mount was attached to the robot flange, the SMR was placed in the monument, and an initial position was measured with the laser tracker. Fastening was then simulated by pressing an Allen key into each of the four screw holes, $S_{1},\;S_{2},\;S_{3},$ and $S_{4}$, with a force of 25 N and taking a measurement for each. The results are shown in Table 8 and the raw data can be found on figshare under the following link: https://doi.org/10.6084/m9.figshare.27604596.v1 (accessed on 20 December 2024). The following equations were used to calculate the maximum shift using the absolute value of the maximum difference between the initial pose, $I_{0}$, and the four measurements, $S_{1},\;S_{2},\;S_{3}$, and $S_{4}$:

(16) $$x_{\max.\;\mathrm{Shift}}=\left|\left|x_{I_{0}}\right|-\max_{i\in \{1,..,4\}}\left|x_{S_{i}}\right|\right|;$$

(17) $$y_{\max.\;\mathrm{Shift}}=\left|\left|y_{I_{0}}\right|-\max_{i\in \{1,..,4\}}\left|y_{S_{i}}\right|\right|;$$

(18) $$z_{\max.\;\mathrm{Shift}}=\left|\left|z_{I_{0}}\right|-\max_{i\in \{1,..,4\}}\left|z_{S_{i}}\right|\right|.$$

Taking the maximum shift into account, the comparison between the baseline (BaseMLT) and the mounting and dismounting of the mount (RepM2) in Equations (13)–(15) can be recalculated with the following equations:

(19) $$\frac{\left|\epsilon_{d_{\mathrm{RepM}2}}-x_{\max.\;\mathrm{Shift}}\right|}{\epsilon_{d_{\mathrm{BaseMLT}}}}=\frac{\left|0.075\;\mathrm{mm}-0.159\;\mathrm{mm}\right|}{0.008\;\mathrm{mm}}=\frac{0.084\;\mathrm{mm}}{0.008\;\mathrm{mm}}=10.5;$$

(20) $$\frac{\left|\epsilon_{\alpha_{\mathrm{RepM}2}}-y_{\max.\;\mathrm{Shift}}\right|}{\epsilon_{\alpha_{\mathrm{BaseMLT}}}}=\frac{\left|0.328\;\mathrm{mm}-0.155\;\mathrm{mm}\right|}{0.048\;\mathrm{mm}}=\frac{0.173\;\mathrm{mm}}{0.048\;\mathrm{mm}}=3.6041\bar{6};$$

(21) $$\frac{\left|\epsilon_{\beta_{\mathrm{RepM}2}}-z_{\max.\;\mathrm{Shift}}\right|}{\epsilon_{\beta_{\mathrm{BaseMLT}}}}=\frac{\left|0.485\;\mathrm{mm}-0.129\;\mathrm{mm}\right|}{0.01\;\mathrm{mm}}=\frac{0.356\;\mathrm{mm}}{0.01\;\mathrm{mm}}=35.6.$$

If the microshifts of the robot during the installation of the mount are taken into account, the factor between the baseline and the repeatability measurement for the *x*-axis hardly changes, the factor for the *y*-axis is reduced by ≈47% and the factor for the *z*-axis is reduced by ≈27%.

Another result of the microshift experiment was the self-healing mechanism of the robot. After 30 min, the position of the robot was almost the same as the initial position. This should be taken into account for more accurate measurements after the mount has been attached.

For a comparison with the baseline measurements using an adhesive bond as an attachment (BaseMAB), only the values of RepM2 are analysed. This approach is justified because both experiments utilised the same monument but differed in their attachment methods. Consequently, only the measurements taken after the mount were completely removed and remounted show variation. The designed mount enhanced the repeatability of experiments by mitigating inaccuracy through the following factors:

(22) $$\frac{\epsilon_{d_{\mathrm{RepM}2}}}{\epsilon_{d_{\mathrm{BaseMAB}}}}=\frac{0.075\;\mathrm{mm}}{0.194\;\mathrm{mm}}\approx 0.387;$$

(23) $$\frac{\epsilon_{\alpha_{\mathrm{RepM}2}}}{\epsilon_{\alpha_{\mathrm{BaseMAB}}}}=\frac{0.328\;\mathrm{mm}}{1.43\;\mathrm{mm}}\approx 0.229;$$

(24) $$\frac{\epsilon_{\beta_{\mathrm{RepM}2}}}{\epsilon_{\beta_{\mathrm{BaseMAB}}}}=\frac{0.485\;\mathrm{mm}}{1.726\;\mathrm{mm}}\approx 0.281.$$

In addition to the comparison with the baseline measurements, a comparison with the eccentricity results is also interesting. The eccentricity radius can be compared with $\epsilon_{\alpha_{\mathrm{RepM}2}}$ and $\epsilon_{\beta_{\mathrm{RepM}2}}$ as they lie on the same plane. The highest eccentricity radius was 0.353 mm (≡0.706 mm diameter), $\epsilon_{\alpha_{\mathrm{RepM}2}}$ was 0.328 mm, and $\epsilon_{\beta_{\mathrm{RepM}2}}$ was 0.485 mm. So, the deviation introduced by the eccentricity diameter was 0.706mm−0.328mm=0.378mm and 0.706mm−0.485mm=0.221mm higher. All the values are the worst-case values. The standard deviation, $s_{N}$, was lower by around 50%.

Combined, the eccentricity and repeatability errors add an inaccuracy of 0.706mm+0.485mm=1.191mm in the worst case. This is something to consider when using the mount for high-accuracy applications as it affects measurements.

Finally, the designed mount is compared with the other attachment methods, which were mentioned in the introduction (Section 1). Table 9 gives an overview for different categories. All methods require a monument or nest for an SMR. From this point on, all methods are cheap or even free. In terms of installation, an adhesive bond is flexible as it can be used almost anywhere. It is quick to install but difficult to remove. Depending on the type of adhesive, a solvent may be required. Magnetic mounting is also very flexible, but the robot arm must be magnetic. The Universal Robots robots, for example, are made out of aluminium, which is not magnetic. The magnetic mount is quick to install and easy to remount. The designed mount of this paper is directly compatible with ISO 9409-1-50-4-M6 flanges. It requires only four screws to install and is also easy to remount. The inaccuracy introduced by eccentricity is only ≈27% and the variation in repeatability is only ≈23% to 39% compared to an adhesive bond and a magnetic mount.

## 5. Conclusions

Despite its potential inaccuracies in eccentricity and repeatability, the designed mount for attaching an SMR to a robot flange offers an easy-to-build, affordable, and accessible solution for robot measurements using laser trackers. It supports the repeatability of research experiments and measurements, improving the traceability of results. It enables research laboratories to verify the results of colleagues and fellow researchers. The success of the mount depends largely on its distribution and use in laboratories. The more users there are, the more comprehensible and reproducible the results based on this measuring instrument will be.

The next steps are to further improve the design to reduce eccentricity and increase repeatability. Other materials will be compared to the 3D-printed mount, such as CNC machined aluminium, steel, or carbon. The mount could be extended to rotate the SMR to keep it aligned with the laser tracker as the robot moves. This could be performed by adding a bearing and changing the design of the mount. Or, the mount could be extended to hold up to four SMR monuments for a six-dimensional perception of the mount with the position and orientation of the robot flange in $x,\;y,\;z,\;R_{x},\;R_{y},$ and $R_{z}$.

## Figures and Tables

**Table 1:** The analysed baseline measurement values for the comparison and validation of the laser tracker and the laboratory settings.

	*x*/mm	*y*/mm	*z*/mm
Mean	3158.43	−556.389	−107.327
Median	3158.43	−556.39	−107.327
Min	3158.428	−556.413	−107.331
Max	3158.436	−556.365	−107.321
$\left|\min-\max\right|$	0.008	0.048	0.01
Standard deviation $s_{N}$	0.0018	0.0088	0.0021

**Table 2:** Analysed repeatability measurement values of adhesive bond used as attachment for comparison.

	*x*/mm	*y*/mm	*z*/mm
Mean	3182.528	−559.358	−106.176
Median	3182.521	−559.36	−106.171
Min	3182.429	−560.064	−107.117
Max	3182.623	−558.634	−105.391
$\left|\min-\max\right|$	0.194	1.43	1.726
Standard deviation $s_{N}$	0.0656	0.4852	0.5248

**Table 3:** Analysed radii of all ten eccentricity measurements.

Measurement Run	Radius $r_{\mathrm{fit}}$/mm
Circle 1_1	0.340
Circle 1_2	0.315
Circle 2_1	0.308
Circle 2_2	0.324
Circle 3_1	0.320
Circle 3_2	0.317
Circle 4_1	0.316
Circle 4_2	0.321
Circle 5_1	0.319
Circle 5_2	0.353

**Table 4:** The analysed repeatability measurement values of the SMR in the monument placement.

	*x*/mm	*y*/mm	*z*/mm
Mean	3158.01	−557.399	−107.311
Median	3158	−557.41	−107.314
Min	3157.996	−557.421	−107.331
Max	3158.067	−557.306	−107.256
$\left|\min-\max\right|$	0.069	0.115	0.075
Standard deviation $s_{N}$	0.02	0.0323	0.0199

**Table 5:** The analysed repeatability measurement values of the mounting and dismounting of the mount.

	*x*/mm	*y*/mm	*z*/mm
Mean	3158.32	−556.618	−107.353
Median	3158.32	−556.678	−107.403
Min	3158.284	−556.73	−107.495
Max	3158.359	−556.412	−107.01
$\left|\min-\max\right|$	0.075	0.328	0.485
Standard deviation $s_{N}$	0.0256	0.1147	0.1748

**Table 6:** Comparison between the expected errors from the technical data sheet of the laser tracker FARO Vantage E and the values of the BaseMLT.

	Data Sheet FARO Vantage E	BaseMLT
$\epsilon_{d}/\mathrm{mm}$	0.018	0.008
$\epsilon_{\alpha }/\mathrm{mm}$	0.035	0.048
$\epsilon_{\beta }/\mathrm{mm}$	0.035	0.01

**Table 7:** A comparison between the BaseMLT, the BaseMAB, the repeatability measurement of the SMR in the monument placement (RepM1), and the repeatability measurement of the mounting and dismounting of the mount (RepM2).

	BaseMLT	BaseMAB	RepM1	RepM2
$\epsilon_{d}/\mathrm{mm}$	0.008	0.194	0.069	0.075
$\epsilon_{\alpha }/\mathrm{mm}$	0.048	1.43	0.115	0.328
$\epsilon_{\beta }/\mathrm{mm}$	0.01	1.726	0.075	0.485

**Table 8:** A display of the microshifts of the robot during the attachment of the mount by comparing the initial position and the positions after simulating fastening the screws.

	*x*/mm	*y*/mm	*z*/mm
Initial position $I_{0}$	3156.944	−556.126	−106.897
Screw 1 $S_{1}$	3157.089	−556.281	−107.015
Screw 2 $S_{2}$	3157.061	−556.274	−107.019
Screw 3 $S_{3}$	3157.103	−556.271	−107.015
Screw 4 $S_{4}$	3157.085	−556.271	−107.026
Max. Shift	0.159	0.155	0.129
Position after 5 min	3156.869	−556,256	−106.995
Position after 30 min	3156.943	−556,140	−106.901

**Table 9:** Comparison between different attachment methods and designed mount in different categories.

	Adhesive Bond	Magnetic Mount	Designed Mount
Cost (every method needs a monument/nest)	Cheap (≈USD 0.5/attachment)	Free	Cheap if a 3D printer is available (≈USD 1.27)
Installation	Flexible, fast, and difficult to remove	Flexible, but the robot arm needs to be magnetic, fast, and easy to remount	Directly compatible with ISO 9409-1-50-4-M6 flanges, attached with four M6 screws, and easy to remount
Eccentricity	1.185mm	1.185mm	0.32mm
Repeatability	$\epsilon_{d}=0.194\mathrm{mm}$	$\epsilon_{d}=0.194\mathrm{mm}$	$\epsilon_{d}=0.075\mathrm{mm}$
	$\epsilon_{\alpha }=1.43\mathrm{mm}$	$\epsilon_{\alpha }=1.43\mathrm{mm}$	$\epsilon_{\alpha }=0.328\mathrm{mm}$
	$\epsilon_{\beta }=1.726\mathrm{mm}$	$\epsilon_{\beta }=1.726\mathrm{mm}$	$\epsilon_{\beta }=0.485\mathrm{mm}$

## Data Availability

The original data presented in the study are openly available in figshare at https://doi.org/10.6084/m9.figshare.27195387.v2 (accessed on 20 December 2024), https://doi.org/10.6084/m9.figshare.28043018.v1 (accessed on 20 December 2024), https://doi.org/10.6084/m9.figshare.28043048.v1 (accessed on 20 December 2024), https://doi.org/10.6084/m9.figshare.27195483.v2 (accessed on 20 December 2024), https://doi.org/10.6084/m9.figshare.27468399.v1 (accessed on 20 December 2024), https://doi.org/10.6084/m9.figshare.28025015.v1 (accessed on 20 December 2024), https://doi.org/10.6084/m9.figshare.27195540.v2 (accessed on 20 December 2024) and https://doi.org/10.6084/m9.figshare.27604596.v1 (accessed on 20 December 2024).

## Abbreviations

The following abbreviations are used in this manuscript:

BaseMAB	Baseline Measurement Adhesive Bond
BaseMLT	Baseline Measurement Laser Tracker and Laboratory Setting
DH	Denavit–Hartenberg
MPE	Maximum Permissible Error
PLA	Polylactide
RepM1	Repeatability Measurement 1
RepM2	Repeatability Measurement 2
SMR	Spherically-Mounted Retroreflector
SVD	Singular Value Decomposition
TCP	Tool Center Point
